# Deciphering roles of protein post-translational modifications in IgA nephropathy progression and potential therapy

**DOI:** 10.18632/aging.205406

**Published:** 2024-01-03

**Authors:** Mengying Sun, Guojuan Shi, Xiaohan Zhang, Chao Kan, Shimin Xie, Weixiang Peng, Wenjun Liu, Peter Wang, Rui Zhang

**Affiliations:** 1 Department of Nephrology, Zhuhai People’s Hospital, Zhuhai Clinical Medical College of Jinan University, Zhuhai, Guangdong 519000, China; 2 Department of Medicine, Zhejiang Zhongwei Medical Research Center, Hangzhou, Zhejiang 310018, China

**Keywords:** IgAN, PTM, ubiquitination, phosphorylation, O-glycosylation

## Abstract

Immunoglobulin A nephropathy (IgAN), one type of glomerulonephritis, displays the accumulation of glycosylated IgA in the mesangium. Studies have demonstrated that both genetics and epigenetics play a pivotal role in the occurrence and progression of IgAN. Post-translational modification (PTM) has been revealed to critically participate in IgAN development and progression because PTM dysregulation results in impaired degradation of proteins that regulate IgAN pathogenesis. A growing number of studies identify that PTMs, including sialylation, o-glycosylation, galactosylation, phosphorylation, ubiquitination and deubiquitination, modulate the initiation and progression of IgAN. Hence, in this review, we discuss the functions and mechanisms of PTMs in regulation of IgAN. Moreover, we outline numerous compounds that govern PTMs and attenuate IgAN progression. Targeting PTMs might be a useful strategy to ameliorate IgAN.

## INTRODUCTION

Immunoglobulin A (IgA) nephropathy (IgAN) is one common glomerulonephritis globally [1]. IgAN, also known as Berger’s disease, is characterized by the accumulation of the glycosylated IgA in the mesangium [2]. IgA plays a crucial role in immune responses, particularly in the mucous membranes, including gastrointestinal and respiratory tracts. IgAN patients might remain asymptomatic for years, while some patients display a wide range of symptoms [3, 4]. Many IgAN patients often present a nephritic syndrome, such as proteinuria, oliguria, and hematuria. Sometimes IgAN patients exhibit gastrointestinal or upper respiratory tract infections [3, 4]. It is known that four-hit hypothesis in IgAN postulates a pathogenesis of this disease: increased production of galactose-deficient IgA (Gd-IgA1) cause the formation of immune complexes with anti-gd-IgA1 IgG or IgA1 antibodies, then deposit in the glomerular mesangium and subsequently result in kidney inflammation and injury [5, 6]. IgAN can be diagnosed by a combination of clinical evaluation, urine tests for proteinuria and hematuria, blood tests for kidney function measurement, a kidney biopsy for confirming the IgA deposits in the glomeruli [7–9]. The treatments of IgAN reduce its symptoms, prevent its complications and retard its progression. The therapeutic strategy of IgAN includes immunosuppressive therapy to suppress the immune system and reduce inflammation, supportive care, dietary modification, blood pressure control and prevention of complications [10–13].

Genetics, epigenetics and environmental factors have been considered to be involved in IgAN development, although the exact molecular mechanisms of IgAN have not been fully elucidated [14–16]. Integration of genomic data with other omics data, such as metabolomics and transcriptomics, can explore the genetic changes of IgAN pathogenesis [17]. In addition, infections contribute to the immune response that contributes to IgA deposition in the kidneys. Epigenetic modifications regulate DNA or chromatin level, resulting in the regulation of gene transcription and protein synthesis [18, 19]. For example, noncoding RNAs regulate IgAN pathogenesis, including miRNAs and lncRNAs [20, 21]. In recent years, post-translational modification (PTM) has been considered to participate in tumorigenesis and IgAN development and progression [22–25]. PTMs change the structure of proteins or chemical properties via removing or adding molecules or functional groups, leading to influencing localization, stability, activity, and interactions with other proteins [26–28]. PTMs include ubiquitination, methylation, acetylation, phosphorylation, glycosylation, etc., [29]. Phosphorylation is known to add a phosphate group to amino acid residues, such as tyrosine, serine, threonine [30]. Ubiquitination means the attachment of ubiquitin to lysine residues on a specific target protein, leading to modulating protein degradation, trafficking and interactions with other molecules [31]. Acetylation means the addition of an acetyl group to the N-terminus or lysine residues of a molecule [32, 33]. Methylation is to add a methyl group to a lysine or arginine of a protein [34]. The attachment of oligosaccharides to serine, threonine or asparagine of a protein leads to glycosylation [35]. Adding small ubiquitin-like modifier (SUMO) proteins to lysine residues in molecules results in sumoylation [36]. Palmitoylation means the addition of a palmitic acid to cysteine residues in a protein [37]. In this review, we will describe the several PTMs in regulation of IgAN development and progression, including sialylation, o-glycosylation, galactosylation, phosphorylation, ubiquitination and deubiquitination. Moreover, we outline multiple compounds to target PTMs and alleviate IgAN progression. Furthermore, future perspectives are discussed to more fully elucidate the functions and mechanisms of PTMs in IgAN. Targeting PTMs could be a potential option for the therapeutic strategy for IgAN patients.

## PTMs regulate IgAN progression

It has been documented that IgA has two isotypes: IgA1 and IgA2. The former IgA1 presents in systemic circulation and mucosal surfaces, while the latter IgA2 is primarily in the mucosal surfaces [38]. In addition, IgA1 and IgA2 are different in the numbers of N-linked carbohydrates in the heavy chain and hinge region (HR). IgA1 displays nine serine and threonine residues in the HR, which has three to six residues with O-linked glycans, whereas IgA2 has no this phenotype [39]. These O-glycan sites can be modified by the addition of N-acetylgalactosamine (GalNAc) to serine or threonine resides. Then, GalNAc residue is extended with a β1,3-linked galactose, forming a core structure [40]. Further, these core O-glycans can be modified by the addition of sialic acid residues, which is known as sialylation. The heterogeneity of IgA1 is governed by the number and structure of O-glycans [40].

## Sialylation

Sialylation is a crucial PTM of proteins via the addition of sialic acid to glycans [41]. ST6GALNAC2 (ST6 N-Acetylgalactosaminide Alpha-2,6-Sialyltransferase 2) is a kind of sialyltransferase that add sialic acids (a type of sugar molecule) to the glycoconjugates. ST6GALNAC2 has been identified as an important gene to regulate the sialylation of Gd-IgA1, contributing to the susceptibility to IgAN [42]. One study showed that ADG haplotype in the ST6GALNAC2 gene involves in the genetic susceptibility in IgAN with a desialylation of IgA1 molecules [43]. Another study suggested that decreased expression of ST6GALNAC2 contributed to reduced sialylation of IgA1 in peripheral B lymphocytes in IgAN patients [44]. Lu et al. reported a correlation between ST6GALNAC2 polymorphism (SNP rs3840858) and IgAN susceptibility [45]. Xie and coworkers found that tonsillectomy increased the expression of ST6GALNAC2 in PBMCs and the plasma IgA1, but it reduced C1GALT1 (core1 β1,3-galactosyltransferase) at mRNA levels in IgAN patients [46].

C1GALT1 is one of glycosyltransferase genes to add galactose to O-glycans for O-glycosylation. An integrated analysis of the transcriptome demonstrated that plasma ST6GAL1 (ST6 β-galactoside a2,6-sialytransferase 1) is increased and correlated with aberrant IgA1 glycosylation in IgAN [47]. ST6GAL1 levels are associated with disease severity of IgAN. Using PBMCs from IgAN patients, recombinant ST6GAL1 attenuated the production of Gd-IgA1 and stimulated the expression of C1GALT1 [47]. Moreover, upregulation of ST6GAL1 promoted sialylation of IgG and reduced the production of cytokines in PBMCs, such as IL-6 and TNF-α. Increased ST6GAL1 was linked to a slower progression of IgAN [48]. Furthermore, IgG sialylation alleviates the formation of Gd-IgA1-containg complexes and reduced inflammation activity and proliferation of mesangial cells in IgAN [49]. Interestingly, ST6GAL1 was observed in human circulating platelets, which could be due to platelets activation to release ST6GAL1 in IgAN [50]. ST6GAL1 polymorphisms affect progression and susceptibility of IgAN patients in a Han population. For example, rs7634389 is associated with hyperuricemia, segmental glomerulosclerosis, renal survival and susceptibility of IgAN. Additionally, rs6784233 ST6GAL1 is correlated to susceptibility of IgAN in a Han population [51].

## O-glycosylation

The functions of IgA can be affected by variations of O-glycosylation, such as innate immunity. It has been shown that increased IgA glycosylation is linked to low levels of IgA and poor outcomes in IgAN patients [52, 53]. One study reported that β1,3-galactosyltransferase and N-acetylgalactosaminyl-transferase 2 were decreased in tonsillar B lymphocytes in IgAN [54]. Zhu et al. found that genetic interactions of ST6GALNAC2 variants and C1GALT1 modified O-glycosylation of IgA1, conferring to IgAN development [55]. C1GALT1 gene has different variants, which are correlated with the genetic susceptibility to IgAN [56–58]. In the following paragraphs, we will discuss how O-glycosylation is regulated in IgAN progression.

### GM130 regulates glycosylation

Loss of the GM130 (Golgi matrix protein 130) contributed to aberrant IgA1 glycosylation in IgAN [59]. Because IgA1 glycosylation is performed in Golgi, reduced GM130 could result in glycosylation deficiency. Indeed, the expression of GM130 was downregulated in tonsil tissues and PBMC in patients with IgAN. It has a negative association between GM130 and Gd-IgA1 production. Downregulation of GM130 enhanced IgA1 O-Glycosylation deficiency due to inhibition of C1GALT1 expression [59].

### miRNAs regulate glycosylation of IgA1

Evidence has implied that microRNAs (miRNAs) participate in IgAN development [60, 61]. It is known that miRNAs belong to short, noncoding RNAs and modulate the gene expression [62]. Clearly, miRNAs have been reported to be involved in various diseases, including cancer [63–65]. One study showed that 37 miRNAs were differentially expressed in IgAN patients compared with healthy controls [66]. Moreover, miR-148b regulated the glycosylation of IgA1 in IgAN. Increased expression of miR-148b was observed in PBMCs of IgAN patients. Overexpression of miR-148b decreased endogenous C1GALT1 mRNA, while silencing of miR-148b elevated C1GALT1 mRNA and protein levels in PBMCs [66]. The expression of miR-148b was negatively associated with C1GALT1 in IgAN patients. Furthermore, miR-148b was associated with Gd-IgA1 levels. Hence, miR-148b is involved in the aberrant glycosylation of IgA1 in IgAN [66]. The high-throughput sequencing data revealed that miR-98-5p was upregulated in the PBMCs of IgAN patients. Mechanistically, miR-98-5p regulated the expression of CCL3 (chemokine ligand 3). Loss of CCL3 modulated the expression of IL-6 and C1GALT1. The treatment of PBMCs with miR-98-5p mimic suppressed the CCL3 and C1GALT1 expression and elevated the expression of IL-6 [67].

Another high-throughput RNA sequencing data suggested that there are 44 differentially expressed miRNAs (34 upregulated, 10 downregulated) in PBMCs of IgAN patients compared with healthy participants [68]. Among 44 miRNAs, 41 of which were linked to IgAN progression. Moreover, the target genes of these miRNAs were enriched in MAPK and PI3K/Akt pathways. Notably, miR-200a-3p, miR-203a-3p and miR-3121-3p might regulate the expression of C1GALT1 [68]. Li et al. reported that inhibition of miR-214-3p alleviated mesangial hypercellularity in IgAN due to upregulation of PTEN and inhibition of JNK/c-Jun pathway, contributing to suppression of proliferation of mesangial cells and attenuation of renal lesions in IgAN [69]. Astragaloside IV inhibited the expression of miR-98-5p and reduced the Gd-IgA1 levels in DAKIKI cells. In addition, overexpression of miR-98-5p could modulate the IgA1 glycosylation by regulation of C1GALT1 [70]. One group reported that let-7b downregulated the expression of GALNT2 in PMBCs of IgAN patients [71]. Another group reported that overexpression of miR-374b increased cell proliferation and enhanced the production of glycosylated IgA1 via targeting PTEN and Cosmc expression in B cells of IgAN [72]. IgAN patients had a low expression of miR-155 level in PMBCs. IgAN patients had low percentages of peripheral blood Treg and Th1 cells, high percentages of Th17 and Th2. The association was identified between miR-155 levels, serum IgA concentration, Cosmc, FoxP3, and IgA1 dys-glycosylation [73]. Li et al. discovered that miR-320 elevated B cell proliferation and accelerated the production of glycosylated IgA1 via targeting PTEN and Cosmc in IgAN [74]. IgAN patients had an upregulation of miR-320 and a downregulation of Cosmc in urinary and renal tissues [74]. Liu and coworkers dissected that miR-630 targeted TLR4 (Toll-like receptor 4) and affected the expression of IL-1β and IL-8 via NF-κB pathway, leading to governing production of underglycosylated IgA1 in the tonsils of IgAN patients [75]. Taken together, miRNAs regulate IgAN development and progression.

### lncRNAs regulate IgA1 glycosylation

lncRNAs have been validated to play an essential role in a variety of diseases, such as cancer [76–79]. Recently, studies have revealed that lncRNAs take part in IgAN development. For instance, Sun et al. found that lncRNA FGD5-AS1 targeted PTEN-involved JNK/c-Jun pathway via sponging miR-196b-5p, contributing to alleviation of childhood IgAN [80]. Shen et al. reported that lncRNA CRNDE enhanced activation of NLRP3 inflammasome in macrophages, including Il-1β, TNF-α and IL-12, and exacerbated the malignant progression of IgAN [81]. CRNDE can bind with NLRP3 and increase the expression of NLRP3. Silencing of CRNDE attenuated the NLRP3 expression at protein level and facilitated TRIM31-induced ubiquitination and degradation of NLRP3 [81]. ICAM-1 related lncRNA (ICR) acts as antisense strand for inhibiting ICAM expression and has been reported to be involved in IgAN and renal fibrosis [82]. IgAN patients had an increased ICR level in renal tissues, which was associated with disease progression. Suppression of ICR by shRNA inhibited the expression of pAkt, mTOR, collagen I and α-SMA in HK-2 cells after TGF-β1-treatment [82]. IgAN patients had a downregulation of lncRNA H19 expression in serum compared with healthy people. Moreover, lncRNA H19 had a protective role for prognosis in IgAN. Higher expression levels of H19 suggested better renal outcome in IgAN patients [83]. LncRNA PTTG3P was upregulated in IgAN samples and urinary of IgAN patients. Overexpression of PTTG3P promoted the expression of Ki-67 and Cyclin D1 and enhanced B cell proliferation as well as triggered production of IL-8 and IL-1β [84]. PTTG3P inhibited the expression of miR-383 in B cells. Consistently, overexpression of miR-383 blocked B cell proliferation and attenuated production of IL-8 and IL-1β. Hence, PTTG3P stimulated B cell proliferation and promoted glycosylated IgA1 production in IgAN [84].

## Galactosylation

Growing evidence reveals that galactosylation involves in IgAN development and progression [85, 86]. Reduced terminal galactosylation of HR O-linked moieties in IgAN was reported, which could link to the pathogenesis of IgAN [85]. Mass spectrometry analysis provided a direct evidence for reduced sialylation and galactosylation of IgA1 Fc O-glycosylated hinger peptides in IgAN, such as GalNAc and Gal, indicating that reduced sialylation and galactosylation of IgA1 might lead to its glomerular deposition [86]. One study showed higher serum Gal-deficient IgA1 levels in IgAN cases and their first-degree relatives, compared with their spouses and normal people, indicating that Gal-deficient IgA1 could be inherited in IgAN patients [87]. A study showed that Chinese patients with IgAN has a lower Gd-IgA1 compared with white people with IgAN. C1GALT1 gene was correlated with Gd-IgA1 levels, and Gd-IgA1 was upregulated in IgAN cases and associated with disease progression. Furthermore, common variation of C1GALT1 regulated Ga-IgA1 levels [88]. Another group found that IL-4 and IL-6 accentuated galactose deficiency of IgA1 through inhibition of C1GALT1 and indirect upregulation of ST6GALNAC2, which blocks galactosylation by C1GALT1, contributing to reduced galactosylation of the O-glycan in IgAN [89]. Evidence confirmed that C1GALT1 expression was reduced and negatively associated with increased expression of Gd-IgA1 in IgAN cases [90]. IgAN patients had a lower expression and activity of β1,3-galactosyltransferase in peripheral B lymphocyte. Therefore, C1GALT1 expression is linked to IgA1 galactosylation in B cells in IgAN [90]. Altogether, IgA1 galactosylation is involved in IgAN progression via C1GALT1.

## Ubiquitination and deubiquitination

**Figure 1:**
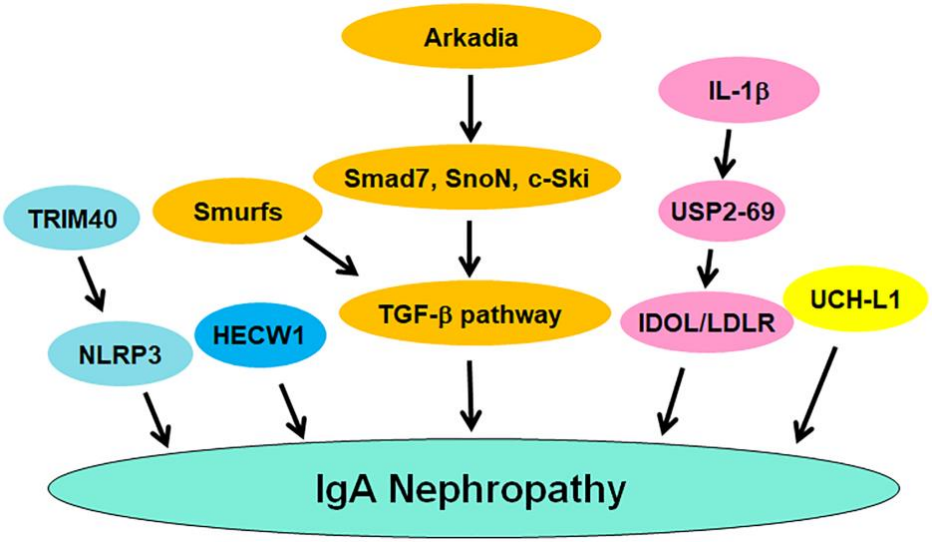
**The role of ubiquitination and deubiquitination of proteins in regulating IgAN pathogenesis.** TRIM40, Smurfs and Arkadia regulate the occurrence and progression of IgAN. USP2-69 and UCH-L1 participate in IgAN progression.

The ubiquitin proteasome system (UPS) is responsible for the protein degradation and maintains protein homeostasis, contributing to governing various cellular processes, including cell cycle regulation, DNA repair, autophagy, apoptosis, differentiation, and proliferation [91, 92]. The UPS includes several main components, such as ubiquitin, ubiquitin-activating enzyme E1, ubiquitin-conjugating enzyme E2, ubiquitin ligase E3, and proteasome [93]. Ubiquitin, a small protein about 8.5 KDa with 76 amino acids, can be covalently attached to the lysine residues of target proteins. This ubiquitination process is performed by E1, E2 and E3 enzymes. Ubiquitin can be activated by E1 enzymes and then transferred to E2 enzymes. The latter conjuncts with E3 enzymes, leading to transfer of ubiquitin to the target proteins [94]. E3 ligases can recognize specific targets for their ubiquitination. Then, ubiquitinated proteins can be recognized and degraded by the proteasome to become smaller peptides and amino acids, which can be recycled [95]. E3 ligases have two main subgroups based on their structures: HECT domain-containing E3 ligases and the RING domain-containing E3 ligases [96]. It is known that E3 ligases play a crucial regulatory role that ensures the proper protein turnover. Dysregulation of E3 ligases have contributed to the development of various diseases [97–101]. Evidence has suggested a link between the ubiquitin proteasome pathway and IgA nephropathy. Herein, we discuss the role of ubiquitination in IgAN progression (Figure 1).

### UCH-L1

UCH-L1 (ubiquitin carboxyl-terminal hydrolase L1), also known as PGP9.5 (protein gene product 9.5) and PARK5, belongs to the UPS and is responsible for degrading and recycling proteins in cells, which maintains protein homeostasis and governs various cellular processes, such as cell cycle, proliferation, apoptosis, autophagy, etc., [102]. UCH-L1 regulates the degradation of the targeted proteins, leading to protein turnover and protein quality control [103]. UCH-L1 is predominantly observed in neurons, and its gene mutations have been found in certain neurodegenerative disorders, such as Parkinson’s disease and Alzheimer’s disease [104–106]. A growing number of works have demonstrated that UCH-L1 is closely related to IgA nephropathy. One study used pre-embedding immunoelectron microscopy approach with gold and HRP to detect the expression level of UCH-L1 in podocytes of glomerulonephritis. This study found the high density of gold particles or DAB in combination with UCH-L1 in cytoplasm and processes of podocytes from IgA nephropathy and lupus nephritis [107]. The level of anti-UCHL1 antibody was elevated in serum of patients with FSGS (focal segmental glomerulosclerosis) compared with IgAN, membranoproliferative glomerulonephritis and membranous nephropathy, indicating that anti-UCHL1 antibody might be a biomarker for diagnosis of FSGS [108]. Moreover, minimal change disease (MCD) patients and steroid-sensitive FSGS had a higher expression of anti-UCHL1 antibody compared with other glomerulopathies, including IgAN. Anti-CD40 antibody was upregulated in steroid-resistant FSGS, implying that anti-CD40 antibody and anti-UCHL1 antibody might be the biomarkers for differential diagnosis and treatment [109].

### Arkadia and Smurfs

Arkadia is a RING-type E3 ubiquitin ligase and has been identified to regulate the TGF-β signaling pathway. One group reported that Arkadia promoted the ubiquitination and degradation of Smad7, SnoN and c-Ski, conferring to activation of TGF-β signaling pathway [110]. Another group found that Arkadia, Smurf2, and c-Ski could be key regulators in TGF-β signaling and serve as potential targets for cardiac fibrosis [111]. It is known that TGF-β1/Smad signaling has a significant role in renal tubular injury and glomerular sclerosis in IgAN children [112]. One study validated that Arkadia activated TGF-β signaling via governing the degradation of Smads, contributing to TGF-β-mediated IgA expression in IgAN [113]. Interestingly, Smurfs E3 ligases exhibited the opposing effects of Arkadia in TGF-β-involved IgA isotype expression [114]. Upregulation of Smurf1 and Smurf2 attenuated GLα promoter activities induced by TGF-β1, and inhibited Smad7 promoter activity. Increased Smurf1 and Smurf2 reduced Smad3/4-induced and Runx3-induced GLα promoter activities. Smurfs blocked TGF-β pathway and inhibited the expression of GLα. Upregulation of Smurf1 reduced TGF-β-involved IgA secretion. Upregulation of Arkadia abrogated the suppressive function of Smad7 on TGF-β-mediated IgA secretion and GLα expression. All in a word, Arkadia degraded Smad7 and promoted TGF-β-mediated IgA secretion, whereas Smurf1 abolished this effect [114].

### USP2-69

USP2-69, an isoform of USP2, has been reported to be involved in the development of various diseases. One group showed that USP2-69 was highly expressed in breast invasive ductal carcinoma. Overexpression of USP2-69 in MCF-7 cells increased proliferation and S phase fraction, promoted the expression of cyclin D1 and reduced the expression of p27 [115]. Another group reported that USP2-69 and USP2-45 can regulate the LDLR (low-density lipoprotein receptor) signaling pathway via interacting with the E3 ligase IDOL (inducible degrader of the LDLR) to promote its deubiquitylation [116]. Because IDOL mediated the ubiquitylation and lysosomal degradation of LDLR, blockade of this process could promote hepatic LDL-cholesterol clearance. Hence, USP2-69 might be useful to regulate hepatic LDL-cholesterol clearance [116]. USP2-69 alleviated meta-inflammatory factors in macrophages and regulated the development of type-2 diabetes [117]. USP2-69 upregulation inhibited the progression of anti-Thy1.1 nephritis in rat [118]. Upregulation of USP2-69 led to inhibition of cell proliferation and ECM deposition, which was accompanied with suppression of Collagen IV, Fibronectin and Ki-67 [118]. USP2-69 was highly expressed in kidney tissues. Compared with normal kidney, USP2-69 expression was elevated in IgAN, lupus nephritis and APGN (acute proliferative glomerulonephritides), which also displayed the higher expression of PCNA (proliferation cell nuclear antigen). IL-1β and anti-thymocyte serum increased the mRNA and protein levels of USP2-69 in the rat mesangial cells, and increased the expression of PCNA and reduced the expression of p27. Hence, USP2-69 could modulate the proliferation of mesangial cells and participate in IgAN and glomerulonephritis [119].

### Other E3 ligases

Tonsillectomy did not affect the activation of innate immunity, pro-oxidative milieu and ubiquitin-proteasome pathways in IgAN patients. The extra-tonsillar MALT (mucosal associated lymphoid tissue) could lead to hyperactivation of innate immunity in IgAN patients with tonsillectomy [120]. NLRP3 expression was upregulated in IgAN patients, which was mainly located in the tubular epithelial compartment. In primary renal tubular cells (HPTC), TGF- β1 treatment induced the expression of NLRP3 at mRNA and protein levels. When ubiquitin-mediated degradation and transcription led to loss of epithelial phenotype in HPTC, NLRP3 expression was diminished. NLRP3 was reduced in abundance due to ubiquitin-mediated degradation in progressive IgAN, suggesting that NLRP3 is associated with outcome in IgAN [121]. TRIM40 E3 ubiquitin ligase suppressed IgA1-mediated proliferation of GMCs (glomerular mesangial cells) via inactivation of NLRP3 inflammasome through induction of ubiquitination of NLRP3 [122]. HECW1 E3 ubiquitin ligase has been reported to be associated with IgA1 glycosylation in IgAN patients. Patients with higher expression of Gd-IgA1 and IgA1 often had a low expression of HECW1. Gd-IgA1 expression levels were negatively associated with HECW1 mRNA expression [123].

## Phosphorylation

Evidence has suggested that the deoxynivalenol (DON) enhanced IgA hyper-elevation and promoted IgA deposition via upregulation of phosphorylation of MAPKs and JNK1/2 in mesangium [124]. Lipopolysaccharide (LPS) and IgA increased the mRNA and protein levels of TLR4 (Toll-like receptor 4) and promoted the phosphorylation of MAPKs in MMC [125]. One study indicated that phosphorylation of ERK was involved in the pathogenesis of IgAN [126]. Another study reported that DON promoted phosphorylation of CREB (cAMP response element binding protein) at Ser-133 and mediated the phosphorylation of ATF1 at Ser-63 in mouse macrophage, which can be suppressed by DHA treatment [127]. DHA consumption attenuated the phosphorylation of Akt and subsequently suppressed the CREB/ATF1 phosphorylation and blocked transcription of IL-6 in mice [127]. Podocytes were cultured with mesangial medium isolated from IgAN patients and exhibited EMT phenotype due to promotion of phosphorylation of Akt [128]. One group showed that suppression of miR-21 blocked fibrogenic activation via upregulation of PTEN expression and downregulation of phosphorylation of Akt in tubular cells and podocytes in IgAN [129]. The phosphorylation of c-Jun was elevated in nuclei of glomerular and tubular cells in several renal diseases, including IgAN. TGF-β triggered the phosphorylation of c-Jun in HK-2 cells, which can be abolished by a JNK inhibitor SP600125 [130]. In IgAN patients, IgA1 immune complexes activated cultured mesangial cells in part via modulation of phosphorylation patterns of three proteins [131]. Inhibition of miR-29b-3p elevated the expression of CDK6 and activated NF-κB pathway via phosphorylating p65, contributing to inflammation in IgAN pathogenesis [132].

BAFF (B cell activating factor) accelerated phosphorylation of p65, Akt and MAPK p38 in mesangial cells via interaction with BAFF-R (BAFF receptor), leading to promotion of cell proliferation [133]. Serum pIgA (polymetric IgA) increased activation of Src and Smad3 phosphorylation as well as nuclear p65 accumulation in human mesangial cells. CTRP3 (complement-C1q TNF-related protein 3) attenuated inflammatory response and mesangial cell activation, resulting in attenuation of IgAN progression [134]. IgAN patients had an increased p53 phosphorylation and STAT3 activation [135]. IgAN patients had an increased expression of glomerular β-1,4-galactosyltransferase. Anti-β-1,4-galactosyltransferase antibodies can inhibit the IgA-mediated phosphorylation of spleen tyrosine kinase and reduce the synthesis of IL-6 in mesangial cells [136]. IL-6 facilitated phosphorylation of STAT3 in the cells from IgAN patients, leading to increased production of Gd-IgA1. This process can be abolished by inhibitors of JNK/STAT signaling [137]. LncRNA lnc-TSI (TGF-β/Smad3-interacting long noncoding RNA) was reported to repress TGF-β-mediated phosphorylation of Smad3 and suppress renal fibrogenesis [138]. Dendrin nuclear translocation caused phosphorylation of JNK in podocytes, promoted apoptosis and regulated focal adhesion. Blockade of dendrin nuclear translocation by suppression of importin-α reduced podocyte loss and prevented glomerulosclerosis in nephropathy [139].

**Figure 2:**
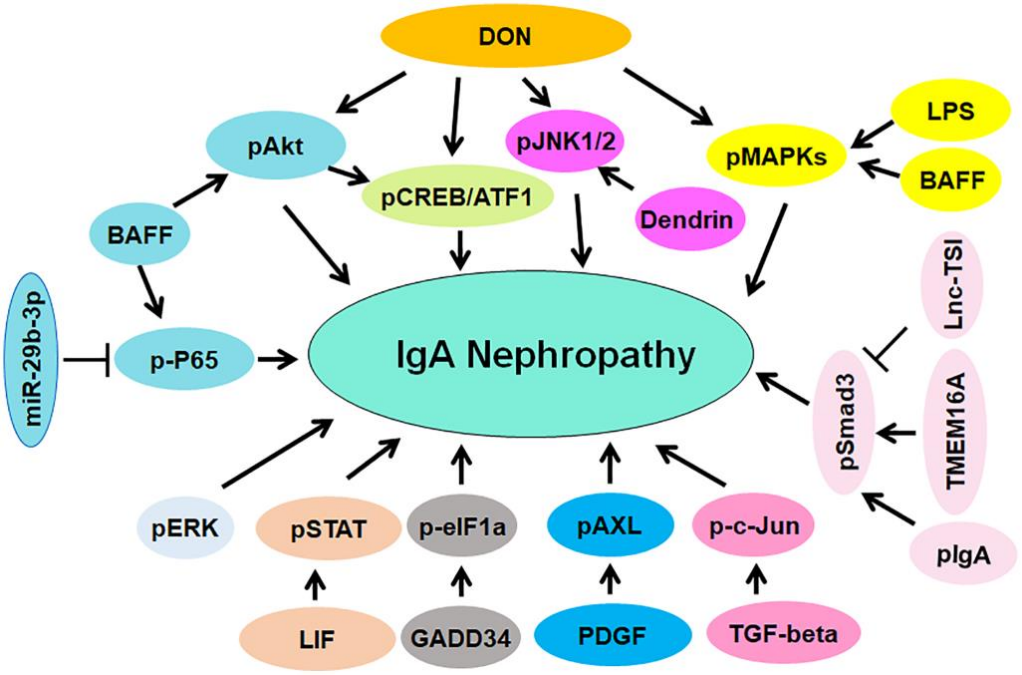
**The role of protein phosphorylation in regulating IgAN pathogenesis.** The phosphorylated proteins are involved in IgAN pathogenesis, including pAkt, p-P65, pJNK1/2, pCREB/ATF, pMAPKs, pSmad3, p-c-Jun, pAXL, pERK, pSTAT and p-eIF1a.

GADD34 expression was elevated in IgAN patients, which was accompanied with increased apoptosis and IgA secretion. Moreover, GADD34 regulated phosphorylation of eIF1a in TMCs (tonsillar mononuclear cells) from IgAN patients [140]. Phosphorylation of STAT1 and STAT3 was increased in IgAN patients. Moreover, STAT1 activation was linked to proteinuria in IgAN patients [141]. Leukemia inhibitory factor (LIF) enhanced Gd-IgA1 production and promoted STAT1 phosphorylation due to activation of Src-family PTKs in the cells from IgAN patients. Consistently, downregulation of STAT1 by siRNA abolished LIF-induced overproduction of Gd-IgA1 [142]. Phosphorylation of glomerular AXL was increased in IgAN patients. Bemcentinib, an inhibitor of AXL, attenuated PDGF-stimulated cell proliferation and inhibited AXL phosphorylation and PDGFR, leading to inactivation of Akt1 and ERK1/2 pathways in IgAN [143]. TMEM16A (transmembrane member 16A), a Ca^2+^-depended chloride channel, was elevated in IgAN patients. Depletion of TMEM16A attenuated TGF-β1-mediated EMT, decreased the expression of Snail1 and reduced the phosphorylation of Smad2/3 and ERK1/2 in HK2 cells, while upregulation of TMEM16A led to the opposite functions. TGF-β1-mediated phosphorylation of Smad2/3 was abrogated by reduction of C1-concentration in HK2 cells. Inhibition of TMEM16A could be a strategy for the treatment of renal fibrosis [144]. The soluble CD22 (sCD22) was decreased in plasma of IgAN patients and positively correlated with SA-IgG (sialic acid-positive IgG). SA-IgG promoted the CD22 phosphorylation in PBMCs and accelerated sCD22 release in cell supernatant, which suppressed the production of proinflammatory cytokines, such as TGF-α, TGF-β and IL-6 [145]. Taken together, protein phosphorylation is critically involved in IgAN pathogenesis (Figure 2).

## Compounds targets PTMs in IgAN

Accumulated evidence has demonstrated that numerous compounds can target protein phosphorylation to regulate the progression of IgAN (Table 1). IgAN rats displayed higher expression of p70S6K and phosphorylation of Akt and S6. Rapamycin, a mTOR inhibitor, reduced the expression of p70S6K and phosphorylation of S6, resulting in reduction of proteinuria, IgA deposition, which protected renal function in IgAN rats. Targeting Akt/mTOR/p70S6K by rapamycin might be a good option for IgAN therapy [146]. Rapamycin increased autophagy and suppressed the phosphorylation of mTOR and S6K1 and reduced the expression of cyclin D1 in IgAN rats [147]. Tris DBA, a palladium complex, was found to ameliorate IgAN via inactivation of NLRP3 inflammasome and promotion of autophagy induced by SIRT1 and SIRT3. Tris DBA treatment in IgAN mice inhibited phosphorylation of ERK, p38 MAPK and JNK, suppressed ROS generation, contributing to improving kidney functions and albuminuria [148]. TwHF (tripterygium wilfordii Hook F), a compound for treating IgAN patients in China, inhibited phosphorylation of JUN and alleviated renal injury in the renal of IgAN mice [149].

**Table 1:** Targeting PTMs by compounds in IgAN.

**Items**	**Targets**	**Functions**	**References**
Rapamycin	mTOR, S6 and S6K1 phosphorylation	Reduction of proteinuria, IgA deposition, protection of renal function	[146, 147]
Tris DBA	Phosphorylation of ERK, p38 MAPK, JNK, ROS generation	Improving kidney functions and albuminuria	[148]
TwHF	Phosphorylation of JUN	Alleviating renal injury in IgAN mice	[149]
Artemisinin	Akt phosphorylation and Nrf2 nuclear translocation	Reduction of fibrosis and oxidative stress	[150]
KF506	Calcineurin, TRPCs, α-SMA, ERK phosphorylation	Improving proteinuria, hematuria, kidney functions	[151]
Zhen-wu-tang	Phosphorylation of NF-κB and IκBα, PPARγ	Protecting podocyte injury	[152]
Fish oil	Phosphorylation of MAPKs and JNK1/2; IL-6	Reduction of DON-induced IgAN	[124]
DHA	Phosphorylation of p38, ERK1/2, JNK1/2; IL-6	Attenuation of DON-mediated IgAN	[153]
Icariin	IKKβ and IκBα phosphorylation; NLRP3; NF-κB	Ameliorating IgAN progression	[154]

**Figure 3:**
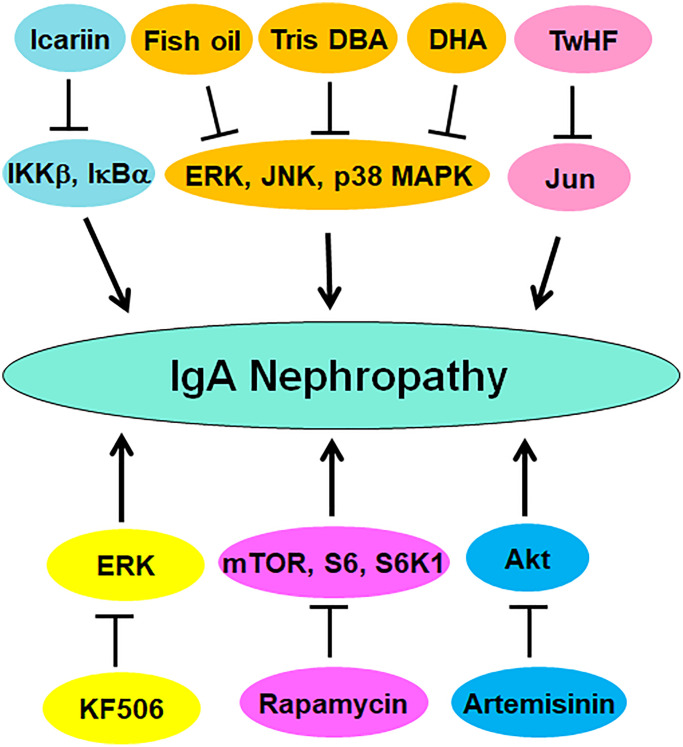
**Multiple compounds target protein phosphorylation to attenuate IgAN progression.** These compounds include fish oil, Tris DBA, DHA, TwHF, Icariin, KF506, artemisinin, and rapamycin.

Artemisinin, a kind of antimalarial drug, was reported to attenuate IgAN by reduction of fibrosis and oxidative stress via modulation of Akt phosphorylation and Nrf2 nuclear translocation [150]. KF506, a calcineurin inhibitor and immunosuppressive compound, was reported to reduce calcineurin, TRPCs and α-SMA and phosphorylation of ERK1/2 in IgAN rats, leading to improving proteinuria, hematuria and kidney functions [151]. Zhen-wu-tang (ZWT), a Chinese medicine, was revealed to suppress the phosphorylation of NF-κB and IκBα and promote the expression of PPARγ in IgAN rats and LPS-induced podocytes, protecting podocyte injury [152]. Fish oil reduced DON-mediated IgAN via suppression of phosphorylation of MAPKs and JNK1/2 and inhibition of IL-6 expression in mice [124]. Similarly, docosahexaenoic acid (DHA) reduced DON-mediated IgAN, IL-6 transcription and phosphorylation of MAPKs in mice, including p38, ERK1/2 and JNK1/2 [153]. Icariin reduced IKKβ and IκBα phosphorylation and blocked the degradation of IκBα, resulting in prevention of nuclear translocation of NF-κB and NLRP3 activation in IgAN rats, suggesting that icariin could ameliorate IgAN progression via modulation of NF-κB and NLRP3 pathways [154]. Altogether, compounds can attenuate the IgAN progression via targeting PTMs (Figure 3).

## CONCLUSIONS

In conclusion, PTMs could play an essential role in the occurrence and progression of IgAN, including O-glycosylation, phosphorylation, ubiquitination and deubiquitination. Multiple compounds target PTMs to attenuate the IgAN progression. Governing PTMs is a promising strategy for the treatment of IgAN. However, several issues need to be mentioned to fully understand the roles of PTMs in the development and progression of IgAN. For instance, sialylation, O-glycosylation, galactosylation, phosphorylation, ubiquitination and deubiquitination have been confirmed to be involved in IgAN progression. SUMO1 accelerated proliferation of mesangial cells via suppression of autophagy in IgAN, indicating that SUMOylation could contribute to the IgAN pathogenesis [155]. It is unclear whether acetylation, palmitoylation and protein methylation are involved in IgAN development. Hence, it is necessary to elucidate the roles of acetylation, palmitoylation and methylation in the progression of IgAN. Besides IgAN, PTMs are critical for lupus nephritis development and progression. For example, UCHL1 involves in lupus nephritis and could be a potential target for lupus nephritis [156]. UCHL1 is regulated by NF-κB in podocytes in glomerulonephritis [157]. In addition, UCHL1 modulates podocyte injury via destroying proteasomes in glomerulonephritis [158]. It is worth noting that E2 enzymes also participate in IgAN development. One group used high-density protein microarrays to measure IgG autoAbs in the normal controls and the serum of IgAN patients. IgG autoAbs, including UBE2W (ubiquitin-conjugating enzyme E2W), matriline 2, protein kinase D1 and DEAD box protein, were upregulated and associated with IgAN in the kidney glomerulus and tubules [159]. Zhou et al. explored the association between SNPs (single-nucleotide polymorphisms) and SLE (systemic lupus erythematosus) in IgAN patients. UBE2L3 was found to be a share gene between IgAN and SLE. Ubiquitin/proteasome-dependent degradation pathway was linked to IgAN and lupus nephritis [160]. Taken together, elucidating the functions and molecular mechanisms of PTMs in IgAN occurrence and progression could provide the novel therapeutic strategies for IgAN patients. Numerous compounds have been reported to attenuate the IgAN progression via targeting PTMs. Hence, modulating PTMs could treat the patients with IgAN in the future.

### Data availability

All data generated during this study are included within this article.
